# The Development of a Multicommand Tactile Event-Related Potential-Based Brain–Computer Interface Utilizing a Low-Cost Wearable Vibrotactile Stimulator

**DOI:** 10.3390/s24196378

**Published:** 2024-10-01

**Authors:** Manorot Borirakarawin, Nannaphat Siribunyaphat, Si Thu Aung, Yunyong Punsawad

**Affiliations:** 1 Faculty of Science and Technology, Nakhon Si Thammarat Rajabhat University, Nakhon Si Thammarat 80280, Thailand; manorot_bor@nstru.ac.th; 2 School of Informatics, Walailak University, Nakhon Si Thammarat 80160, Thailand; nannaphat.sir@mail.wu.ac.th; 3 Department of Mathematics, University at Buffalo, State University of New York, Buffalo, NY 14260-2900, USA; sithuaun@buffalo.edu; 4 Informatics Innovative Center of Excellence, Walailak University, Nakhon Si Thammarat 80160, Thailand

**Keywords:** brain–computer interface, electroencephalography, event-related potential (ERP), tactile stimulation, Emotiv EPOC Flex

## Abstract

A tactile event-related potential (ERP)-based brain–computer interface (BCI) system is an alternative for enhancing the control and communication abilities of quadriplegic patients with visual or auditory impairments. Hence, in this study, we proposed a tactile stimulus pattern using a vibrotactile stimulator for a multicommand BCI system. Additionally, we observed a tactile ERP response to the target from random vibrotactile stimuli placed in the left and right wrist and elbow positions to create commands. An experiment was conducted to explore the location of the proposed vibrotactile stimulus and to verify the multicommand tactile ERP-based BCI system. Using the proposed features and conventional classification methods, we examined the classification efficiency of the four commands created from the selected EEG channels. The results show that the proposed vibrotactile stimulation with 15 stimulus trials produced a prominent ERP response in the Pz channels. The average classification accuracy ranged from 61.9% to 79.8% over 15 stimulus trials, requiring 36 s per command in offline processing. The P300 response in the parietal area yielded the highest average classification accuracy. The proposed method can guide the development of a brain–computer interface system for physically disabled people with visual or auditory impairments to control assistive and rehabilitative devices.

## 1. Introduction

Many countries have drawn attention to the need to enhance the physical and mental conditions of people with disabilities by developing policies and guidelines to reduce inequality [1]. Moreover, developing assistive and rehabilitative technologies is a crucial solution. Assistive technology, including assistive devices, prosthetic devices, and related intelligent systems, is another essential aspect for improving the quality of life of people with disabilities [2]. Existing assistive technology for people with disabilities supports all disability levels, such as people with multiple disabilities or those who need specific assistance [3]. The development of assistive technology to serve patients with severe disabilities is challenging. Currently, many studies focus on the application of biomedical signals in human–computer/machine interfaces (HCIs/HMIs), particularly brain–computer interfaces [1,2,3]. BCIs measure brain signals and translate them into commands to control devices such as electric wheelchairs, mechanical arms, and electrical appliances [4,5].

Electroencephalography (EEG) is widely used in brain signal acquisition techniques for developing brain–computer interface systems [1,2,3] because it is safe and easy to use. Event-related potentials (ERPs) that rely on stimuli to cause changes in the brain have been widely used for brain–computer interfaces [6], such as visual, audio, and tactile stimulation [7], the appropriateness of which depends on the remaining ability of the disabled person to perceive. Somatosensory stimulation to produce EEG responses or somatosensory-evoked potentials (SEPs) can be divided into transient somatosensory-evoked potentials (TSEPs) [4,5], tactile P300, which is the neurophysiological response to a tactile stimulus occurring rarely and recognized as target in a random sequence of stimuli, characterized by a positive peak around 300 ms after the stimulus onset [8], and steady-state somatosensory-evoked potentials (SSSEPs) [5]. Vibration and electrical stimulation devices are used as tactile stimuli in BCI systems. Vibration devices can generate vibration with a mechanical frequency using motors and piezoelectric sensors. Electrical stimulation devices can generate and emit electric signals with different waveforms through stimulation electrodes.

In research on tactile BCI systems, Pokorny et al. [5] studied static tactile stimulation by installing vibration motors on both fingertips with a vibration frequency of 237 Hz, alternating between the left and right sides. The results showed that the average accuracies of the SSSEP and P300 methods were both 50.7% and the hybrid SSSEP and P300 methods had an average accuracy of 48.6% and 55.5%, respectively. As an example of a tactile BCI for assistive control, Kaufmann et al. [9] proposed a BCI-controlled wheelchair that utilized tactile ERPs. They applied vibrotactile stimulation at four different locations: the left thigh, right thigh, abdomen, and lower neck. The results of the experiment, which involved a virtual wheelchair navigation task, indicate an average accuracy of 85.8% and a selection time of 27.2 s. Halder et al. [10] introduced a BCI system based on tactile stimulation to control a mobile platform inside and outside a laboratory. The average accuracy values obtained inside and outside the laboratory were 72% and 61%, respectively. Tactile BCIs have been employed to enhance motor imagery (MI) in rehabilitation systems. For example, Zhang et al. [11] used an EEG phase-dependent closed loop to enhance an MI-based BCI by placing it on the index finger of the non-dominant hand. This method helps modulate the mu rhythm and makes the subjects concentrate more on imagery. The tactile fatigue under closed-loop stimulation was significantly lower than that under continuous stimulation. Yao et al. [6] used vibrations to stimulate the skin on the left and right sides to enhance the MI features. Moreover, Grigorev et al. [7] proposed a neurofeedback training system using vibrotactile to improve the MI response.

Table 1 shows a tactile P300-based method for control and rehabilitation that utilizes electric and vibration stimulations [12,13,14,15,16,17,18,19,20]. Regarding the fingertip tactile stimulation paradigm, Yajima et al. [12] proposed a tactile P300 paradigm for people with poor learning ability or difficulty in maintaining attention. They provided wearable tactile stimulus gloves using five vibration motors with random stimulus patterns at the fingertips. The results indicated an average classification accuracy of 80%. Savić et al. [20] demonstrated an electrotactile stimulus of the right forearm’s radial and medial nerves with a control paradigm based on a tactile attention task to elicit sERP. The average classification accuracy ranged from 75.1 to 88.1%. The results demonstrate that the proposed electrotactile stimulus paradigm may be used for assistive and rehabilitation applications. Li et al. [15] demonstrated electrical tactile stimulation of the fingertip using an electrical muscle stimulation device. The average classification accuracy was 79.81%, which is similar to that of vibration motors. Similarly, Chu et al. [17] observed electrical and vibration stimulations at the finger pad and wrist locations. The results showed an average classification accuracy of 94.88% for electrical stimuli and 95.21% for vibration stimuli, which were higher than those of the fingertip location. Furthermore, Guger et al. [14] employed vibrotactile stimulation on the left and right wrists of healthy controls and patients with ALS to elicit ERP phenomena. The results revealed that paradigms utilizing non-VEPs and MI for EEG-based BCIs can communicate with patients in a completely locked-in state (CLIS). Nevertheless, the results of electrical stimulation were similar to those of vibration stimulation. Therefore, vibration stimulation is a popular safety technique for tactile BCI systems.

Kodama et al. conducted a study on vibrotactile stimulation in different parts of the body [13]. They used a tactile plate to provide full-body spatial vibrotactile stimuli (full-body BCI (fbBCI)) in areas such as the arms, shoulders, waist, and legs for a multicommand BCI. The experimental results showed that the system achieved an average accuracy of 98.18% and a real-time accuracy of 53.67%. Furthermore, Chen et al. [15] compared tactile stimulation with and without concurrent visual attention and found that the average accuracy of tactile stimulation with concurrent visual attention was 90.91%, which was higher than the 62.73% accuracy without concurrent attention.

Mao et al. [18] focused on skin friction to develop vibrotactile stimuli and paradigms. They used five vibrators with a silk-stim paradigm (SSP) and a linen-stim paradigm (LSP) to stimulate the left palm, right palm, abdomen, and left and right ankles. The experimental results revealed that the average accuracies of the SSP and LSP were 64.50% and 75.50%, respectively. Moreover, Eidel et al. [19] investigated the potential training factors for both pre- and post-sessions to assess the robustness of the tactile P300 BCI. They used a C-2 tactor, a small actuator that vibrates against the skin, to provide a physical stimulus with randomized stimulation of the left and right lower abdomen and neck. The experimental results revealed that the accuracy ranges from 79.2% to 92.0%.

According to previous studies, tactile ERP-based BCIs can be helpful in control, communication, and rehabilitation [20,21]. Vibrotactile stimulus patterns can be utilized for tactile stimulation and counting the number of stimuli can help focus attention on the target [17,20,22]. An eccentric rotating mass (ERM) vibration motor is an inexpensive device that can be used to generate vibrotactile stimuli and produce high efficiency in paradigms with different stimulus areas [13,16]. In this study, we developed and verified a low-cost wearable vibrotactile stimulus for wrists and elbows based on bone conduction [10] with the proposed feature of a tactile ERP-based BCI system. The results of this study verify the effectiveness of employing a BCI system in offline processing that uses tactile stimulation responses as a proposed approach for application in individuals with physical disabilities, especially those with auditory and visual impairments and lost sensation in the lower limbs.

## 2. Materials and Methods

**Figure 1:**
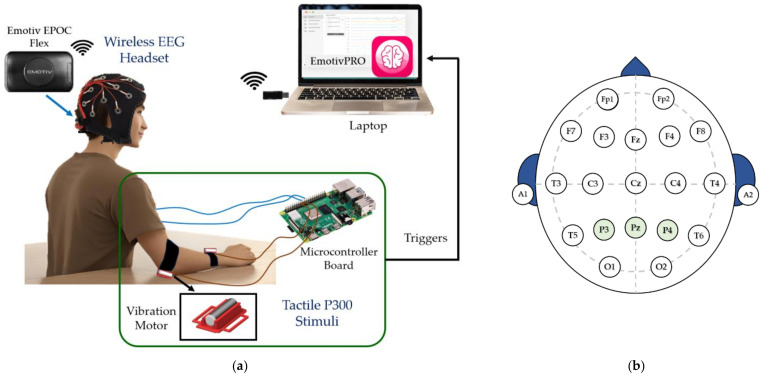
Tactile ERP-based BCI system using a wearable vibrotactile stimulator: (**a**) Components of the proposed tactile BCI based on vibrotactile stimulation. (**b**) Electrode placement for 19 channels based on the 10–20 system and selected electrodes.

Tactile stimulation can trigger sensory perception processes and alter the electrical activity of the somatosensory cortex, affecting the sense of touch [17]. In this study, we designed a strap that provides vibrotactile stimulation and demonstrated a BCI system based on tactile stimuli using EEG signals from the contralateral somatosensory cortex [19]. This system aims to help paralyzed individuals with visual and auditory impairments in control and rehabilitation applications [20]. A tactile BCI system includes tactile stimulation techniques, EEG acquisition, signal preprocessing, feature extraction and classification methods, command translation, and practical applications. The components of the proposed tactile ERP-based BCI system consisted of an EEG machine, vibrotactile stimuli, and a computer, as shown in Figure 1a.

### 2.1. EEG Signal Acquisition

We used the wireless EEG device EPOC Flex [23] from EMOTIV (https://www.emotiv.com (accessed on 16 July 2024)). This device features flexible traditional EEG headcap systems that are designed to minimize the setup time. Electrical brain potentials were measured using saline electrodes and saline-soaked felt pads at a sampling rate of 128 Hz. The data were recorded using the Emotiv Pro software (ver. 3.1.3). The elicited ERP response (in either sensory domain) involves an ensemble of distributed neural networks, including frontal and temporal–parietal brain areas associated with attention and memory processing, thus cortical and subcortical brain regions [8]; in addition, the ERP scalp distribution typically presents maxima over the midline electrodes (Fz, Cz, and Pz) due to the large involvement of different neural populations, and usually without significant lateralization effects as experimentally reported [24]. The reference electrode was placed at the A1-A2 position. We focused on the brain responses from F3, F4, Fz, C3, C4, Cz, P3, P4, and Pz to validate the proposed tactile stimulus patterns for practical BCI systems that use only a few electrodes. Data processing and analyses were conducted using MATLAB (MathWorks) (ver. R2021a). We used a 0.1 Hz to 30 Hz bandpass digital filter to reduce power line noises and eliminate motion artifacts of the stimulus signals using the EEGLAB toolbox (ver. 2023) [25]. For each trial, the filtered signals were segmented at intervals of 200 ms before the onset to collect the baseline and 500 ms after the onset to collect ERP components utilizing the ERPLAB toolbox (ver. 8.10) [26].

Twelve healthy volunteers, consisting of seven females and five males with an average age of 27 years (±3.6), participated in the experiments. All participants had normal tactile acuity and showed no current or past indications of brain disorders, mental disorders, somatosensory nerve symptoms, or migraines. Patients with auditory neurological complications were excluded. Following a review of the documentation, all participants provided written informed consent. The signed consent forms, without personal identification, were kept confidential. All research involving human subjects was approved by the Office of the Human Research Ethics Committee of Walailak University (WU-EC-IN-2-205-66) on 15 September 2023, in accordance with the Ethical Declaration of Helsinki [27], the Council for International Organizations of Medical Sciences [28], and World Health Organization guidelines [29].

### 2.2. Proposed Vibrotactile Stimulation

**Figure 2:**
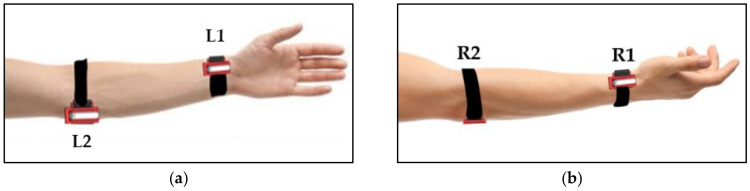
Vibrotactile stimulation positions: (**a**) left elbow and left wrist positions; (**b**) right elbow and right wrist positions.

According to previous studies [12,13,14,15,16,17,18,19], a strap vibrotactile stimulator utilizes a vibration motor based on an eccentric rotating mass (ERM) (7 × 25 mm specification size) to produce a vibration pattern that provides a tactile stimulus. A Raspberry Pi 3 board produced a vibration frequency of 150 Hz (within the frequency range for alerts in personal devices) along with the onset trigger for each tactile stimulus position. The output was amplified using a TLP281 opto-isolation board to drive the ERM vibration motor. The vibrating force was similar to that of a mobile phone’s haptic alert. The ERM motors were attached to a plastic mount on a strap that could be adjusted to fit the participant’s wrist, as shown in Figure 1a (green box). We also verified the proposed tactile stimulus paradigm for a BCI system using four vibrotactile stimuli to investigate the duration and number of trials of the tactile stimulus for P300 elicitation. As shown in Figure 2, the four positions for tactile stimulation were as follows: (1) left wrist (L1), (2) right wrist (L2), (3) left elbow (L3), and (4) right elbow (L4).

**Figure 3:**
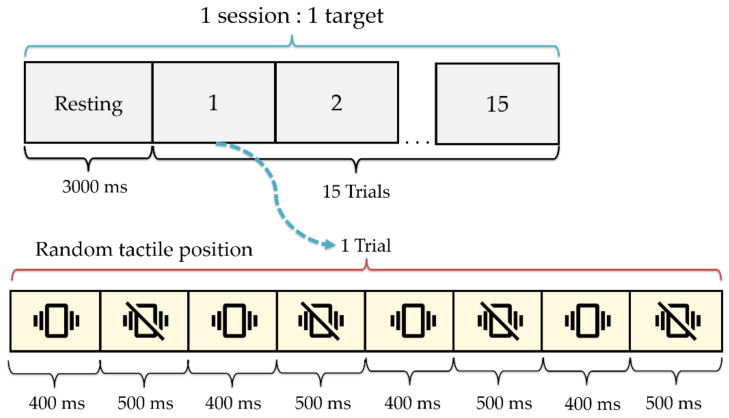
The experimental paradigm of a single session for vibrotactile stimulus using random tactile positions.

The experimental paradigm is shown in Figure 3. The paradigm started with a 3000 ms resting state for EEG baseline collection. Each session involved 15 trials of a tactile stimulus. Each trial lasted 3.6 s and included four vibrotactile stimuli, each lasting 400 ms, presented in randomized order at four locations (L1, L2, R1, and R2) separated by 500 ms pauses. Each participant performed only the target position, following the sequence shown in Table 2, which included 12 targets (sessions) in three rounds. Before proceeding to the next round, the participants were allowed to rest for 5 min.

### 2.3. ERP Feature Selection and Classification

**Figure 4:**
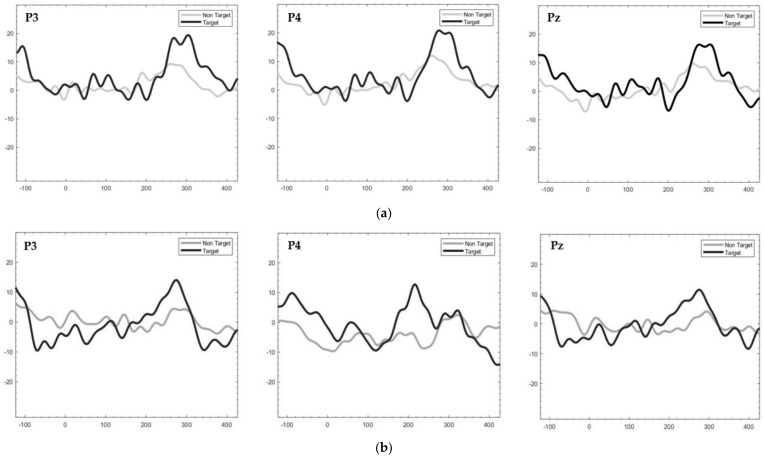
Examples of Grand-averaged ERPs over 10 trials from selected EEG electrodes from the L2 target: (**a**) Grand-averaged ERPs of Participant 3; (**b**) Grand-averaged ERPs of Participant 8.

We observed ERP features by creating the averaged EPRs. The black lines represent the target responses, and the gray lines represent the non-target responses. We found that the processed EEG data obtained from all participants revealed ERP responses when comparing the target and non-target results, as shown in Figure 4. The averaged EPRs of Participant 3 showed a clear P300 component for all EEG channels in the black plot in Figure 4a, which occurred approximately 200 to 400 ms after the onset of the stimuli. However, the ERPs of Participant 8 (Figure 4b) showed a lower P300 response than Participant 3, which was detected approximately 100 to 300 ms after the onset. The results reveal that the distinct P300 components from the selected EEG electrodes, P3, P4, and Pz, via the proposed vibrotactile stimulus were similar to the previous tactile ERP-based BCI [21].

In a previous study [11,12], the P300 component was utilized for ERP feature extraction and as a parameter to identify targets. The segmented EEG signal for each electrode in Section 2.1 compared the average ERP for each target and non-target trial. The maximum amplitude of the average ERP value was recorded in the datasets to predict the target for each participant.

Linear discriminant analysis (LDA) is a popular method for P300 classification [30], with stepwise linear discriminant analysis (SWLDA) being particularly notable for its use of forward and backward stepwise analyses of feature parameters. SWLDA can effectively extract robust features and make accurate predictions for BCIs based on ERP responses [31]. The most correlated features are selected based on statistical significance to predict the targeted command [32]. Conventional techniques using features from the amplitude and latency of N200 and P300, along with SWLDA, can achieve high efficiency in both offline and online testing [33,34]. The SWLDA process begins without feature parameters in the model and proceeds as follows:

(1) Forward analysis adds an input feature parameter to the model, which is then evaluated using an F-test to update the model by adding an input feature $x_{j}$ to the model using the following hypotheses: $H_{0}\;:\;\beta_{j=0}$ (the added feature $x_{j}$ is not significant); $H_{1}\;:\;\beta_{j\neq 0}$ (the added feature $x_{j}$ is significant). If the *p*-value is less than 0.001, the variable is added. The F-test can be obtained via Equation (1) as follows:

(1) $$F=\frac{\left({RSS}_{reduced}{-RSS}_{full})/(p_{full}{-p}_{reduced}\right)}{{RSS}_{full}/({n-p}_{full})}$$

where ${RSS}_{reduced}$ is the residual sum of squares of the reduced model (without $x_{j}$);$\;{RSS}_{full}$ is the residual sum of squares of the full model (without $x_{j}$);$\;p_{full}$ is the number of parameters in the full model; $p_{reduced}$ is the number of parameters in the reduced model; and n is the number of observations.

(2) Backward analysis removes the least significant input feature parameters (*p* > 0.015).

(3) Linear regression is used to include or exclude feature parameters for the prediction of the target label. The model can be examined using Equation (2) as follows:

(2) $$y=\beta_{j}+\sum_{j=1}^{p}\beta_{j}x_{j}+\varepsilon$$

where y is a dependent variable (prediction); $\beta_{j}\;terms\;are$ coefficients; $x_{j}$ terms are feature parameters; and ϵ is the error term.

(4) The training ERP dataset was used to establish the weights of the discriminant model. For a given feature ($x_{j}$), the weight $\left(w_{j}\right)$ determines the contribution of each feature in predicting the target, which can be computed using the training dataset as follows:

(3) $$w_{j}=\frac{Cov(x_{j},\;y)}{Var(x_{j})}$$

(5) The test ERP dataset was computed using the feature weights to distinguish the target and determine the maximum classification score.

### 2.4. Experiments

**Figure 5:**
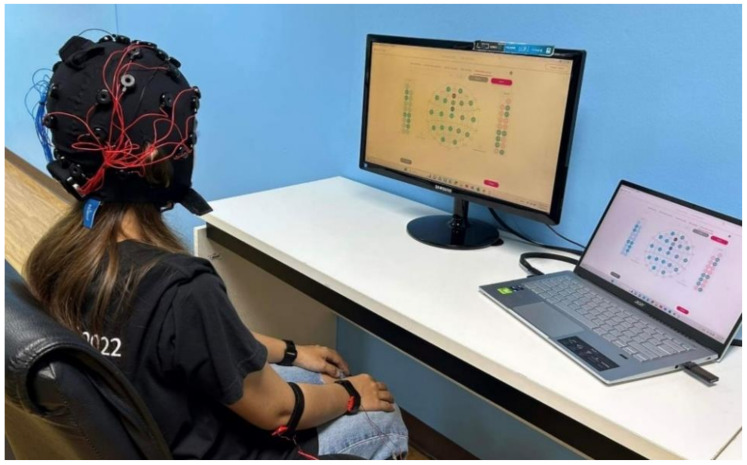
Experimental setup.

The experiment was conducted in a quiet environment, as shown in Figure 5. Before recording the EEG signals, each participant was prepared by fitting them with an electrode cap and ensuring the accurate placement of the electrodes by following the 10–20 system (Figure 1b). The EEG signal quality was checked to ensure good quality. Furthermore, the proposed wearable vibrotactile stimulator with an adjustable strap was installed to ensure the participant’s comfort and ability to perceive the vibration. Subsequently, the participants were briefed on the tactile stimulus experimental paradigm, as shown in Figure 3. Each participant was instructed to focus on the vibrotactile stimulus in the target position to generate a command by following the sequence outlined in Table 2.

The effectiveness of the SWLDA algorithms was verified by using the ERP dataset. To prevent overfitting, we employed three-fold cross-validation with three experimental sessions. In the first step, the classifiers were trained on the data from the first and second sessions and then tested for each participant in the third session. Three stimulus trial conditions were established for each EEG electrode: 5 trials with 480 training and 240 testing observations; 10 trials with 960 training and 480 testing observations; and 15 trials with 1440 training and 720 testing observations. Next, the classifiers were trained in two sessions and tested consecutively in the subsequent session. The effectiveness results are presented for all three stimulus trial conditions with different EEG electrodes in Section 3.1, and the extract results are reported in Section 3.2.

## 3. Results

### 3.1. Observation of Trial of Use and EEG Electrodes for Vibrotactile Stimulus Pattern

**Figure 6:**
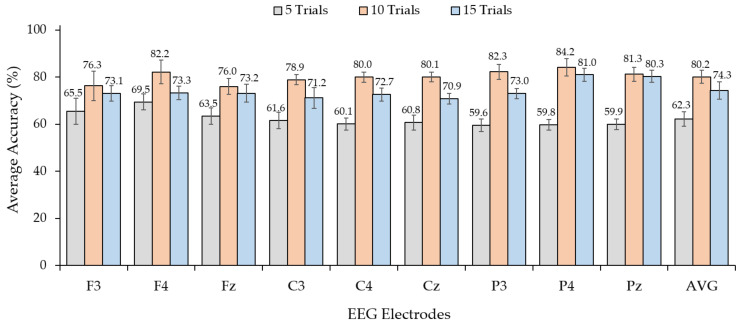
The average classification accuracy corresponding to different positions of electrodes and the number of trials of the vibrotactile stimulus.

For the ERP-based brain–computer interfaces (BCIs), the number of stimulus trials affected the ERP response. The number of stimulus trials is directly correlated with the information transfer rate (ITR), which is crucial for the efficiency of BCI systems. Therefore, we validated the number of trials for the real-time tactile ERP-based BCIs. Figure 6 shows the results from the selected EEG electrodes, which provided an average classification accuracy of >60% for all stimulus trials. We found that the average classification accuracy ranged from 59.6% to 69.5% for five stimulus trials (18 s per command), from 76.0% to 84.2% for 10 stimulus trials (36 s per command), and from 70.9% to 81.0% for 15 stimulus trials (54 s per command). Experiments with more trials yielded a higher accuracy than experiments with fewer trials. Nevertheless, the accuracy may decrease with more trials because of fatigue. We found that using 10 trials for the stimulus resulted in an average classification accuracy of 80.2% for all the electrodes. The EEG signal from channel P3 achieved a maximum average accuracy of 84.2% for 10 trials and 80.3% for 15 trials. Additionally, the EEG signal from the P3 and Pz channels in the parietal area achieved high average classification accuracies of 82.3% and 81.3%, respectively, for 10 trials. The EEG from channel F4 in the frontal area, which influences attention, also achieved an average classification accuracy of 82.2%. 

For statistical analysis, the result dataset was checked for normality and testing. The Shapiro–Wilk test indicated that the average classification accuracy of each stimulus trial and electrode dataset was normally distributed. We then performed a two-way ANOVA to compare the effect of both the number of stimulus trials and electrode positions. A two-way ANOVA revealed that the main effect of the number of stimulus trials was statistically significant (F(2, 297) = 622.364, *p* < 0.001), indicating a substantial effect of the number of stimulus trials on the average classification accuracy. Similarly, electrode positions also had a significant effect on average classification accuracy (F(8, 297) = 2.148, *p* = 0.031), suggesting that changes in electrode positions contribute to variations in the average classification accuracy. Furthermore, the interaction effect between the number of stimulus trials and electrode positions was significant (F(16, 297) = 2.151, *p* = 0.007), implying that the combination of these factors influences the average classification accuracy beyond their individual effects. A post hoc test was used to compare the average classification accuracies differences between the number of stimulus trials and between electrode positions. According to the Bonferroni test, the mean average classification accuracy for 10 stimulus trials was significantly higher than for 5 and 15 trials. Furthermore, the Bonferroni test revealed that the average classification accuracy mean of electrode position P4 was significantly higher than that of seven other electrode positions (C3, C4, Cz, P3, F3, F4, and Fz) (*p* < 0.001). However, it was not significantly higher than Pz. Additionally, the average classification accuracy mean of Pz was significantly higher than that of five other electrode positions (C3, C4, Cz, P3, and Fz) (*p* < 0.001), and not significantly lower than P4 and F4.

Based on these results and related brain areas, we employed the EEG signals from the P3, P4, and Pz electrodes and 10 stimulus trials to observe each target using the vibrotactile stimulus pattern tactile ERP-based BCI system.

### 3.2. Observation of Stimulus Position for Vibrotactile Stimulus Pattern

Table 3 shows the maximum average classification accuracy from the P3, P4, and Pz electrodes. The proposed vibrotactile stimuli at the wrist ranged from 72.80% to 85.04%. For the elbow, the average accuracy ranged from 78.47% to 85.34%. The highest classification accuracy for the right wrist was 80.19%, which was higher than that for the left wrist. Conversely, the highest classification accuracy for the right elbow was 81.68%, which was higher than that for the left elbow. Overall, the average accuracy for the elbow stimulus was higher than that for the wrist stimulus. These results indicate that the vibrotactile stimulus at the elbow can produce a high average classification accuracy for all participants. For some participants, both stimulus patterns produced similar classification accuracies for each stimulus position. In addition, the average classification accuracy for all targets and participants was 80.13%, which is close to the previous tactile BCI systems using the arms area stimulus [18,20], thereby supporting the proposed vibrotactile stimulus pattern.

Moreover, we used classification metrics, including precision, recall, and F1-score, to evaluate the classification. Precision represents the probability of correctly predicted actions out of the total predicted actions, while recall represents the probability of correctly predicted actions out of the actual actions. The F-measure is a balanced measure that combines precision and recall. Table 4 shows the model effectiveness based on the PS, RC, and F1-score given ERP features from electrodes P3, P4, and Pz. The results revealed that the proposed feature could support the model for four target predictions. According to the PS and F1-score results, the model yielded around 80%. The model produced a high RC score for all targets with similar false negative (FN) rates. However, the PS of L1 was the lowest, and a high false positive (FP) rate may have occurred from the L2 stimulus position. This suggests that the proposed methods can be applied to tactile ERP-based BCI systems in real time.

We used a statistical method to analyze the result, as presented in Table 3. A one-way ANOVA was performed to compare the effect of four stimulus positions on average classification accuracy across 12 subjects (*n* = 12), as presented in Table 3. Firstly, we checked the data normality and equality of variances. The Shapiro–Wilk test of normality was conducted to determine whether the average classification accuracy of each stimulus position dataset was normally distributed. The results indicate that we failed to reject the null hypothesis for the average classification accuracy of each stimulus position dataset (*p* > 0.05) and concluded that the data were normally distributed. Consequently, we conducted a one-way ANOVA to determine the effect of stimulus positions. A one-way ANOVA revealed a significant effect of the stimulus position on average classification accuracy (F(3, 44) = 8.366, *p* = 0.0001). Further analysis using the Bonferroni post hoc test indicated that the mean average classification accuracy of the left wrist was significantly lower than that of the right wrist (*p* = 0.001), significantly lower than that of the left elbow (*p* = 0.025), and significantly lower than that of the right elbow (*p* = 0.0002). However, there were no significant differences between the mean average classification accuracy of the right wrist and the left elbow (*p* > 0.05) or between the right wrist and right elbow (*p* = 0.712). Similarly, there were no significant differences between the mean average classification accuracy of the left and right elbow (*p* > 0.05).

**Figure 7:**
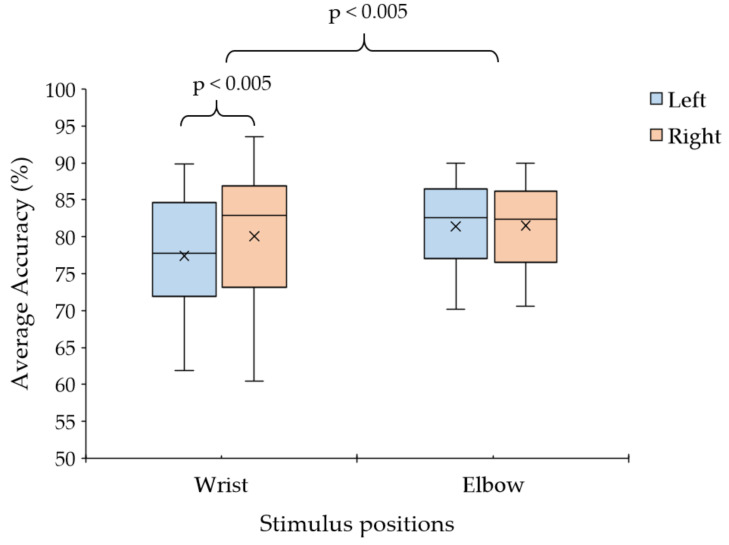
Average classification accuracy between the left and right sides of wrist and elbow positions of the vibrotactile stimulus. (Note: × represents the mean value).

In addition, we used a paired *t*-test for multiple comparisons between the stimulus positions for post hoc tests. We applied the Bonferroni correction, which adjusts the significance level by dividing the original alpha level (0.05) by the number of comparisons made to avoid type I. According to Figure 7, the paired *t*-test (*n* = 12) demonstrated a significant difference in the mean average classification accuracy between the left wrist and right wrist (*p* = 0.0036) and between the left wrist and left elbow (*p* = 0.0094). There was no statistically significant difference in mean average classification accuracy between the left and right elbow (*p* = 0.6632) or between the right wrist and left elbow (*p* = 0.3657). Moreover, the paired *t*-test (*n* = 24) revealed a significant difference between the left and right sides (*p* = 0.0218) and between the wrist and elbow positions (*p* = 0.0013). The tactile stimulus with the proposed wearable vibrotactile stimulator at the right achieved a higher average classification accuracy than the left. The vibrotactile stimulus at the elbow achieved a higher average classification accuracy than the wrist positions.

## 4. Discussion

To evaluate the effectiveness of the proposed wearable vibrotactile stimulator for tactile ERP-based BCIs, we used the SWLDA method to detect the ERP responses. EEG electrodes placed in the parietal areas (P3, P4, and Pz), similarly to those used in previous vibrotactile BCI systems [16,17,18,19,22], produced apparent ERP features (P300), as shown in Figure 4. However, the proposed vibrotactile stimulation required more than 10 stimulus trials using the proposed feature and classification algorithms to achieve high efficiency offline, resulting in acceptable accuracy for the multicommand BCI. For a computational time, the classifier takes 3.84 s to train, 0.48 s to test, and 11.52 s to validate, for a total time of 15.84 s. In addition, we studied the impact of the vibrotactile stimulation position. The results of the experiment indicate that using a low-cost wearable vibrotactile stimulator to stimulate the elbow and wrist areas elicited ERP responses and achieved similar accuracy to previous studies using vibrator devices [18]. This has the potential to increase the number of commands that can be utilized. Furthermore, positioning the elbow can lead to greater efficiency compared to stimulating the wrist area. The proposed wearable vibrotactile stimulator and stimulus patterns can be utilized in practical tactile ERP-based BCI systems for control and rehabilitation applications.

However, the proposed vibrotactile stimulus pattern for the BCI has certain limitations. First, users undergo a training session and receive guidance on how to pay attention to the vibrotactile target. Second, creating each command for the BCI is time-consuming, resulting in low ITR and fatigue. This issue must be addressed in real-time BCI systems. For practical use, it is recommended that a command be used to turn the vibrotactile stimulator on or off. Additionally, we recommend performing additional validations as follows:

(1) Conducting a usability test to perform comparisons among different BCI techniques such as motor imagery (MI) and auditory BCIs;

(2) Employing more targets with different positions, such as the ankle and knee, to further investigate the system performance and user cognition;

(3) Developing a prototype for a wearable vibrotactile stimulator for flexible use through a wireless device.

(4) Verifying the feasibility of implementing the proposed BCI system in a real scenario with impaired participants is essential for practical use.

## 5. Conclusions

In this study, we verified the vibrotactile stimulus pattern to observe the classification accuracy of the proposed feature and classification with different stimulus trials and electrodes. We found that the vibrotactile stimulus pattern required at least 10 stimulus trials (36 s per command) in offline processing and could be used for tactile BCIs. Furthermore, tactile stimulus positions were evaluated. The elbow stimulus yielded a higher classification accuracy than the wrist. Finally, the proposed low-cost wearable vibrotactile stimulator can be used in real-time BCI systems for control and communication to aid in the daily activities of quadriplegic patients with visual and auditory impairments. Moreover, the wearable vibrotactile stimulator can be implemented in tactile-based BCI systems to rehabilitate post-stroke and paralyzed patients [20]. In future studies, we plan to validate the subjects’ ability to maintain attention via ERP responses with a larger number of subjects and verify the practical use of the proposed wearable vibrotactile stimulator in control applications.

## Figures and Tables

**Table 1:** Research studies on the tactile stimulus for the P300-based brain–computer interface (BCI) for applications of control and rehabilitation.

Author	Proposed Methods	Tactile Stimulus	Stimulus Areas	Electrodes	Result(s)
Yajima et al. (2015) [12]	A tactile glove fingertips’ stimulator for a BCI	Five vibrotactile transducers	Fingertips	Cz, CPz, P3, P4, C3, C4, CP5, and CP6,	Average accuracy is 80.0%
Kodama et al. (2016) [13]	A tactile P300 BCI using full-body spatial vibrotactile stimuli (full-body BCI (fbBCI))	Six spatial vibrotactile stimulus patterns	Arms, shoulders, waist, and legs	Cz, Pz, P3, P4, C3, C4, CP5, and CP6	Average accuracy of 98.18%, whereas the real-time accuracy is 53.67%
Guger et al. (2017) [14]	Vibro-tactile P300 and a motor imagery brain—computer interface for LIS patients	Vibrotactile stimulation with 2 and 3 tactors (VT2 and VT3)	VT2: Left and right wrists VT3: Left wrist, right wrist, and shoulder	Fz, C3, Cz, C4, CP1, CPz, CP2, and Pz	Health subject: mean accuracy of 100% in VT2, 93% in VT3 LIS patient: mean accuracy of 76.6% in VT2, 63.1% in VT3
Li et al. (2019) [15]	An online P300 BCI based on somatosensory stimulation paradigm	Electrical muscle stimulation device	Fingertips	Fz, C3, Cz, and C4	Average accuracy is 79.81%
Chen et al. (2020) [16]	Visual attention on tactile P300 BCI for users who only need to focus their attention on a single-target stimulus within a stream of tactile stimuli	Five vibrotactile stimulator (g.VIBROstims)	Left wrist, right wrist, abdomen, left ankle, and right ankle	Fz, FC1, FC2, C3, Cz, C4, CP3, CP1, CP2, CP4, P3, Pz, P4, and Oz	Average accuracy of 90.91% for VA and 62.73% for NVA
Chu et al. (2021) [17]	A novel tactile stimuli P300 paradigm for people with less learning ability or difficulty in maintaining attention	Electric and vibration stimulators	Finger pads and the wrist	C3, C4, CP1, CP2, Cz, F3, F4, FC1, FC2, Fz, P3, P4, POz, and Pz	An average accuracy of 94.88% for electrical stimuli and 95.21% for vibration stimuli
Mao et al. (2021) [18]	The effects of skin friction on tactile P300 BCI performance	Five vibrators with silk-stim paradigm (SSP) and linen-stim paradigm (LSP)	Left palm, right palm, abdomen, left ankle, and right ankle	Fz, FC1, FC2, C3, Cz, C4, CP3, CP1, CP2, CP4, P3, Pz, P4, and Oz	Average accuracy of 64.50% for SSP and 75.50% for LSP
Eidel, and Kübler (2022) [19]	To determine the potential training factors pre/post and assess the robustness of the tactile P300 BCI	Vibrotactor devices (C-2 tactor)	Right and left thigh, abdomen, and lower neck	Fz, FC1, FC2, C3, Cz, C4, P3, Pz, P4, O1, Oz, and O2	Accuracy in the range of 79.2–92.0%
Savić et al. (2023) [20]	A novel electrotactile stimulus with a control paradigm based on tactile attention tasks and sERP	Electrotactile stimuli with two stimulation channels	Radial styloid and medial epicondyle for right forearm	C3, Cz, C4, CP3, Pz, and Fp1	Average accuracy in the range of 75.1 to 88.1%

**Table 2:** The target sequence of the vibrotactile ERP stimulus for the experimental task.

Session	1	2	3	4	5	6	7	8	9	10	11	12
Target	L1	R1	R2	L2	R2	L1	R1	R1	L2	L1	L2	R2

**Table 3:** The average classification accuracy from the selected electrodes under four targets for all participants.

Participants	Average Classification Accuracy (%)			
	L1 (Left Wrist)	R1 (Right Wrist)	L2 (Left Elbow)	R2 (Right Elbow)
1	76.59	78.66	82.23	80.79
2	74.79	80.47	80.14	84.74
3	78.39	85.04	78.71	81.18
4	80.81	82.06	78.47	82.75
5	80.82	79.01	83.10	80.93
6	78.88	80.89	84.17	83.16
7	78.08	79.75	78.77	80.79
8	74.72	79.65	80.66	80.14
9	73.76	79.28	83.86	78.97
10	81.34	80.71	81.00	81.01
11	72.80	76.64	84.36	80.41
12	77.95	80.13	79.41	85.34
Mean ± SD	77.41 ± 2.89	80.19 ± 2.03	81.24 ± 2.23	81.68 ± 1.91

**Table 4:** The classification matrix under four targets for all participants.

Targets	Precision (PS)	Recall (RC)	F-Measure (F1)
L1	0.78	0.94	0.85
R1	0.82	0.95	0.88
L2	0.83	0.95	0.89
R2	0.84	0.95	0.89

## Data Availability

The data presented in this study are available upon request.
